# An all-arthroscopic surgery technique for snapping popliteal tendon syndrome: A case report and literature review

**DOI:** 10.1097/MD.0000000000031347

**Published:** 2022-11-04

**Authors:** Ji Hyun Ryu, Se-Won Lee, Dong Hwan Lee

**Affiliations:** a Department of Orthopedic Surgery, Yeouido St. Mary’s Hospital, College of Medicine, The Catholic University of Korea, Seoul, Republic of Korea.

**Keywords:** all-arthroscopic surgery, lateral knee snapping, popliteus tendon, snapping popliteus tendon syndrome

## Abstract

The incidence of snapping popliteus tendon syndrome, a type of lateral knee snapping, is not high, so making an accurate diagnosis is difficult. A proper treatment following an accurate diagnosis is essential for improvement. Very few cases have been reported of its treatment.

**Patient concerns:**

An 18-year-old male patient had experienced painful popping in the lateral part of the knee during knee flexion for 3 years before his hospital visit.

**Diagnoses:**

Snapping popliteus tendon syndrome.

**Interventions:**

The patient underwent an all-arthroscopic surgery. Tendon debulking and tissue debridement around the popliteus tendon was conducted, but the snapping did not resolve. The enlarged tubercle was excised through an arthroscopic procedure using a burr, and the surgery was finished after confirming that snapping was resolved.

**Outcomes:**

Full range of motion (ROM) was recovered 6 weeks after surgery and the snapping did not recur.

**Lessons:**

Snapping popliteus tendon syndrome is a disease that is hardly recognized due to its low prevalence and difficulty in diagnosis, and it requires close observation of the patient before surgery. The location of the tenderness and the snapping occurrence must also be carefully identified. Our procedure is an entirely arthroscopic technique; as it has the prominent advantage of a speedy recovery and easy rehabilitation, it could also be helpful to set treatment standards for this disease in the future.

## 1. Introduction

Lateral knee snapping is a rare disease; patients who experience lateral knee snapping may hear a popping sound or feel a snapping sensation during a specific motion and the condition is often accompanied by pain. There are various causes of lateral knee snapping, including iliotibial band friction, biceps femoris tendon snapping, popliteomeniscal fascicle tear, popliteus tendon snapping, etc; as the symptoms of this condition are not specific, it is often difficult to differentiate it from other diseases.^[1,2]^ The incidence of snapping popliteus tendon syndrome, a type of lateral knee snapping, is not high, so making an accurate diagnosis is difficult. A proper treatment following an accurate diagnosis is essential for improvement. Accordingly, caution is required for the diagnosis and treatment of this condition. There have been 6 case reports of snapping popliteus tendon syndrome so far,^[3–8]^ and the last report was the case reported by Krause et al in 2008. Recently, a case of cyamella in the popliteal tendon, which causes snapping and discomfort, has been reported, but that was a special case and could be considered a disease with a different pathophysiology.^[9]^ Arthroscopy has been frequently used for the diagnosis of snapping popliteus tendon syndrome, but the treatment was often conducted as an open procedure. In this case report, the diagnosis and treatment of snapping popliteus tendon syndrome were all conducted by arthroscopic procedures, and it was successfully treated. Accordingly, the results of the case study of snapping popliteus tendon syndrome are reported here.

## 2. Case description

**Figure 1.**
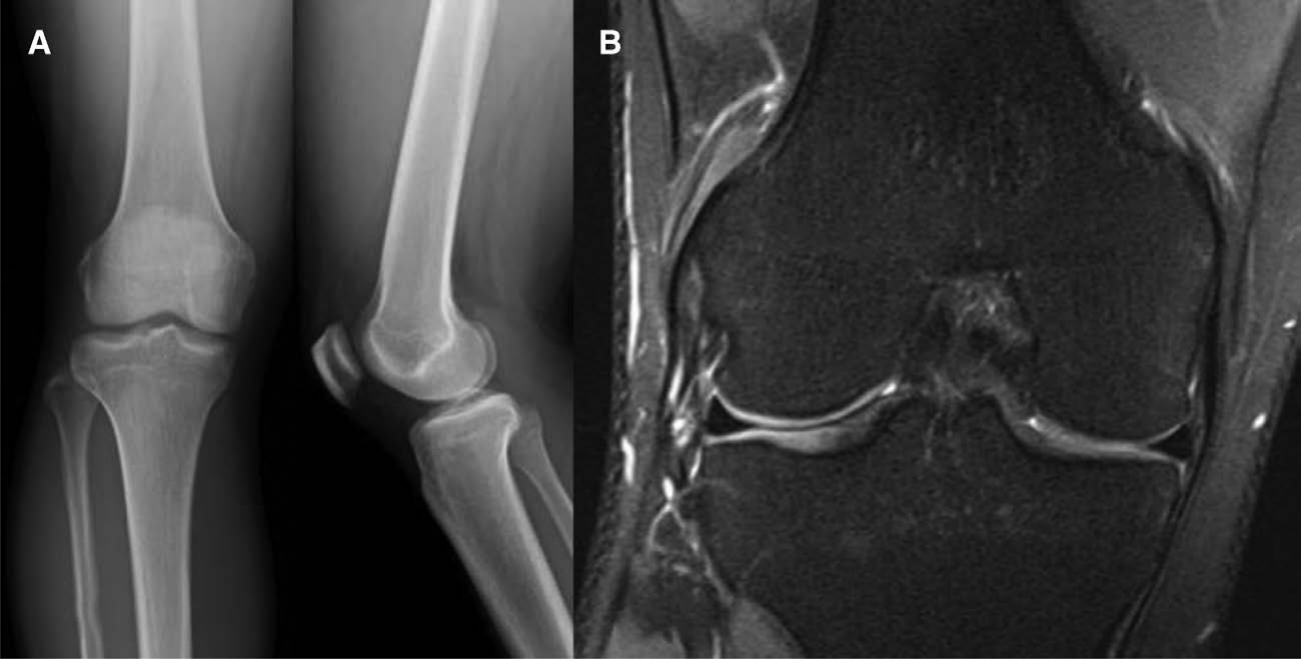
(a) Plain knee radiograph before surgery (b) MRI coronal image: there are no abnormal findings on either the radiograph or the MRI. MRI = magnetic resonance imaging.

**Figure 2.**
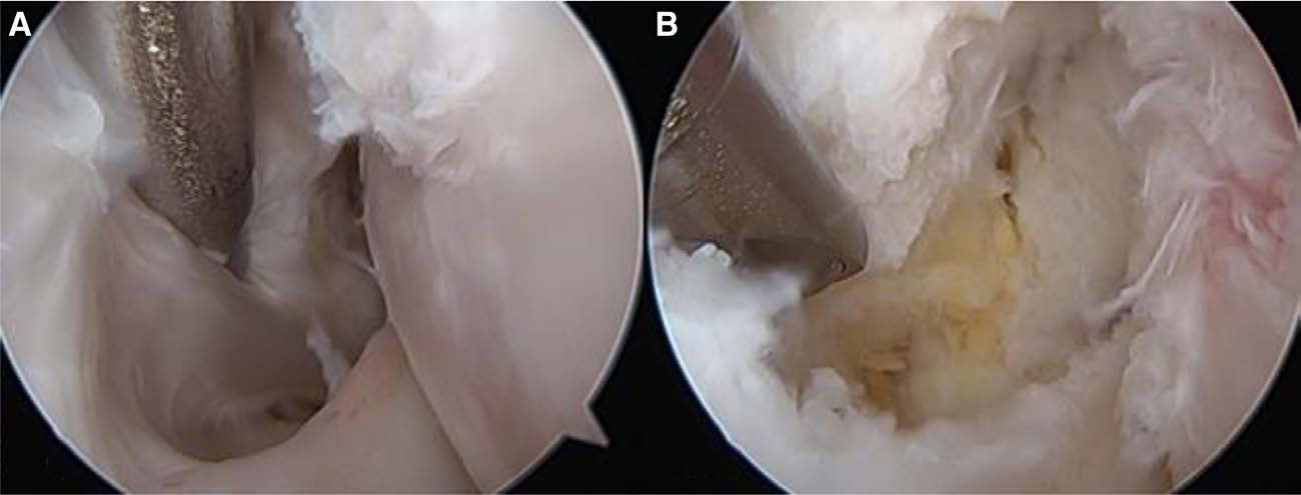
(a) Arthroscopic findings of the popliteal tendon. Abnormal hypertrophied, fibrotic tissues are seen around the popliteal tendon. (b) After the debulking procedure, the snapping was not resolved.

**Figure 3.**
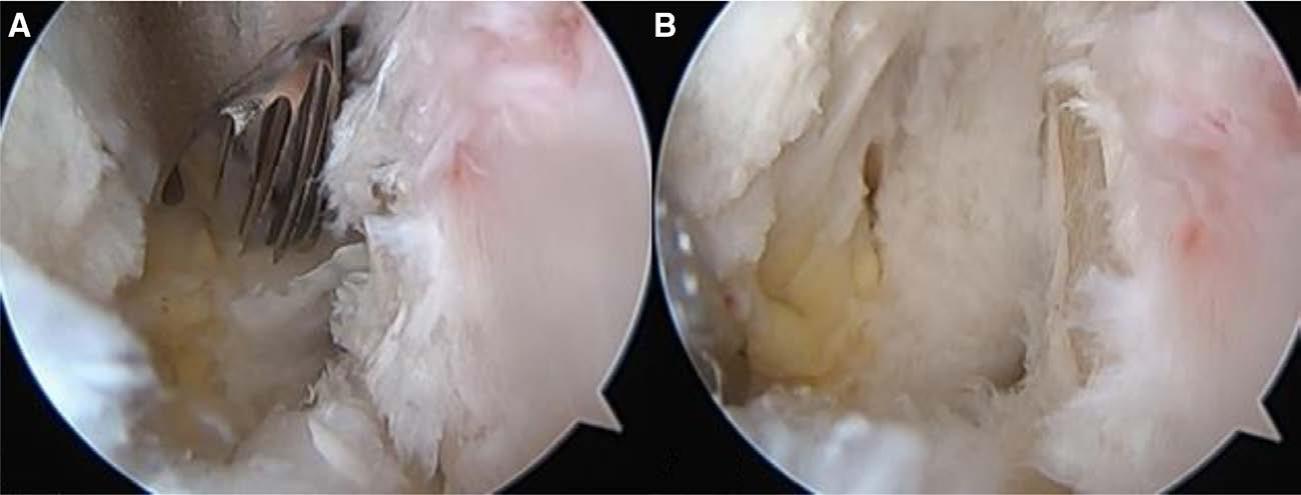
(a) The enlarged tubercle is excised using a burr. (b) The arthroscopic findings after the tubercle excision. Snapping of the popliteal tendon was resolved after excision.

An 18-year-old male patient had experienced painful popping in the lateral part of the knee during knee flexion for 3 years before his hospital visit. He reported no history of trauma. The patient said that he had been a football player until high school and that when the painful popping got worse, he stopped playing football. However, his symptoms had not improved. No other specific findings were shown on physical examination, and we heard a clear popping sound during knee flexion and extension (see Video 1, Supplemental Video 1, http://links.lww.com/MD/H743, this video was taken before surgery, and a clear popping sound can be heard). No specific findings were shown on plain radiography, and there were no abnormal findings of the meniscus or cartilage on magnetic resonance imaging (MRI). Inflammatory changes around the popliteus tendon may have been responsible for the popping, but the etiology was not clear (Fig. 1). The patient’s condition did not improve during 2 weeks of conservative treatment, so diagnostic arthroscopy was planned. Arthroscopic findings showed no meniscus lesions, no cartilage lesions, and no loose body. Fibrotic hypertrophy was visible near the popliteus tendon, and snapping over a tubercle under the popliteus sulcus was also noted. Tendon debulking and tissue debridement around the popliteus tendon was conducted, but the snapping did not resolve (Fig. 2). The enlarged tubercle was excised through an arthroscopic procedure using a burr, and the surgery was finished after confirming that snapping during flexion and extension was resolved (Fig. 3) (see Video 2, Supplemental Video 2, http://links.lww.com/MD/H745, this video shows the arthroscopic procedure used during surgery, which was an all-arthroscopic technique). After surgery, range of motion (ROM) and quadriceps strengthening exercise were performed while the patient received adequate pain control; full ROM was recovered 6 weeks after surgery and the snapping did not recur (see Video 3, Supplemental Video 3, http://links.lww.com/MD/H746, this video was taken after surgery. No popping sounds were audible during knee flexion-extension movement).

## 3. Discussion

The popliteus muscle originates from the posterior surface of the proximal tibia, passes through the popliteal hiatus as a tendon, and attaches to the lateral femoral condyle. It is a tendon with a fairly wide footprint on the lower anterior surface of the lateral epicondyle and the proximal half of the popliteal sulcus with a complex structure that is connected to the lateral meniscus by popliteomeniscal fascicles as it passes through the popliteal hiatus.^[10,11]^ The popliteus muscle is known to play an essential role in the knee posterolateral anatomic relationship. According to LaPrade et al, it is located anterior to the popliteal sulcus from full extension to 112º flexion and is located in an anteriorly subluxated position. Additionally, it is engaged into the popliteal sulcus from 112º flexion to full flexion.^[12]^

**Figure 4.**
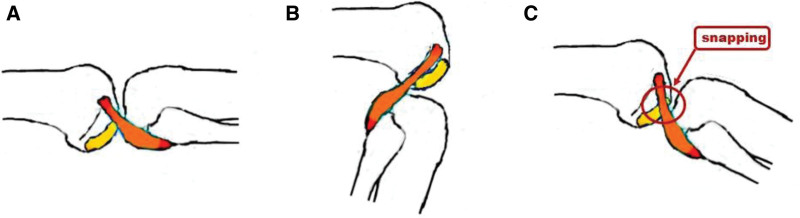
Gross anatomy of the popliteal tendon, sulcus, and tubercle. The orange-colored structure is the popliteal tendon, and the yellow-colored structure is the tubercle. (a) The popliteal tendon is placed anterior to the tubercle with the knee in full extension. (b) The popliteal tendon is fully engaged with the sulcus with the knee in flexion over 112º. (c) Snapping between the popliteal tendon and the tubercle occurs mostly at 20º to 40º of flexion. The red circle indicates where the snapping occurs.

Although it is slightly different across individuals, engagement occurs into the popliteal sulcus after a certain angle of flexion. Before engagement, snapping sometimes occurs in the tubercle located below the popliteal sulcus in the mid-flexion state. Case reports have shown that snapping occurs mostly at 20º to 40º of flexion (Fig. 4). If one hears a popping sound or feels pain due to severe snapping, the condition is diagnosed as snapping popliteus tendon syndrome, as in this case. Although the pathophysiology of this condition has not been clearly identified, based on many reports, it occurs mainly in patients with intense knee activity, like athletes. Therefore, the popliteal tendon snapping seems to get worse due to inflammatory changes, tissue hypertrophy, and other situations characterized by repeated friction on the bulky tubercle and tendon.

Snapping popliteus tendon syndrome is a rare disease; only 6 cases have been reported so far. It is difficult to diagnose as no specific findings were shown on plain radiography or MRI. Cooper et al conducted a physical examination to confirm popliteus snapping when performing flexion-extension ROM while applying varus stress in the “figure of four” position, and all 6 patients showed positive findings.^[3]^ Mariania et al also reported positive findings for Cabot’s sign, which is almost the same test.^[6]^ However, this type of physical examination has a somewhat low specificity because it may not be clearly differentiated from pathologic signs of other lateral compartment structures, such as fibular collateral ligament, or medial compartment structures. Furthermore, it is difficult to discuss the sensitivity of the test due to the insufficient number of cases. Therefore, its use is considered limited.

As shown above, snapping popliteus tendon syndrome is a condition that is difficult to diagnose based on imaging tests or physical examination alone; the use of arthroscopic examination is helpful to diagnose and treat this condition. In this case, we performed an arthroscopic examination and could confirm that snapping occurs, and the snapping was treated with all arthroscopic procedure. Debulking of the hypertrophied fibrotic tendon and surrounding tissues was carried out, and the enlarged tubercle was excised using a burr because the snapping had not resolved after debulking.

Various treatments have been introduced in the case reports so far, and there were many cases of operation with the open procedure after diagnostic arthroscopy. In these 6 case reports, surgeries could be classified into 3 procedures: popliteal tendon release or debulking, tenodesis with or without sulcus deepening, and tubercle excision (Table 1).

**Table 1:** A review of reported snapping popliteal tendon syndrome.

	Cases (n)	Individual treatment options
Crites et al (1998)	1	Open excision of the edge of the sulcus (open tubercle excision)
Cooper et al (1999)	6	Conservative treatment in 4 patients; 1 required a re-operation later because of a failed initial result.
		Tenodesis with FCL in 1 patient
		Tendon resection in 1 patient
Mcallister et al (1999)	2	Sulcus deepening and tenodesis of bilateral knees in 1 patient
Gaine et al (2002)	3	Arthroscopic osteophyte excision in 2 patients Arthroscopic tendon resection in 1 patient
Mariani et al (2005)	3	Diagnostic arthroscopy and conservative treatment in 1 patient Sulcus deepening and tenodesis in 2 patients
Krause et al (2008)	1	Tenodesis with FCL and tubercle excision

FCL = fibular collateral ligament.

The way to resect the entire popliteal tendon can completely resolve the occurrence of snapping, but it has the disadvantage of removal of the function of the popliteus. This may induce posterolateral and rotatory instability and may affect meniscus withdrawal during flexion.^[13,14]^ Some cadaver studies have shown that isolated popliteal tendon resection did not induce posterolateral instability.^[15]^ However, clinically, resection may affect some of the various functions of popliteus. Therefore, it should be considered as the last method when snapping is not resolved even after treatment with other methods.

Techniques such as tenodesis with fibular collateral ligament or tenodesis after sulcus deepening procedure cannot be performed with arthroscopic surgery alone. Since invasive open surgery is required, this procedure has the disadvantages of a poor recovery after surgery, prolonged rehabilitation, and the risk of complications, such as adhesions.

In the case of tubercle excision, some cases required open excision, but excision with a sufficient margin could be made with all arthroscopic procedures alone, as was done in the present study. In 2 cases, Gain et al also showed that it could be treated through arthroscopic osteophyte excision. However, in the cases of Gain et al, the mean age of the patients was slightly higher, and the condition was not associated with the athletic lifestyle. A clear osteophyte was visible on both plain radiography and arthroscopic examination.^[5]^ These may have been cases in which the locations of snapping were similar to other snapping popliteal tendon cases but the pathogenesis was slightly different. In our case report with a younger patient, there was no osteophyte on plain radiography or MRI. It is significant to mention that the condition could be treated through tubercle excision and debulking of hypertrophied tissue with arthroscopy alone even in a case with a young patient without a more protruded osteophyte.

It is difficult to determine whether popliteal tendon release or debulking, tenodesis with or without sulcus deepening, or tubercle excision is the best method for treatment, as only a few cases have been reported until now. However, if this condition can be treated with a less invasive treatment, the less invasive method should be performed.

## 4. Conclusion

Snapping popliteus tendon syndrome is a disease that is hardly recognized due to its low prevalence and difficulty in diagnosis, and it requires close observation of the patient before surgery. The location of the tenderness and the snapping occurrence must also be carefully identified. Based on this, it is important to accurately diagnose and resolve the pathology of popliteus tendon without any misdiagnosis. Our procedure is an entirely arthroscopic technique; as it has the prominent advantage of a speedy recovery and easy rehabilitation, it could also be helpful to set treatment standards for this disease in the future.

## Author contributions

**Conceptualization:** Dong Hwan Lee.

**Formal analysis:** Ji Hyun Ryu.

**Methodology:** Se-Won Lee, Dong Hwan Lee.

**Supervision:** Se-Won Lee, Dong Hwan Lee.

**Writing – original draft:** Ji Hyun Ryu.

**Writing – review & editing:** Dong Hwan Lee.

## Supplementary Material


